# Tumor Microenvironment-Mediated Resistance in HER2-Positive Breast Cancer: Recent Advances and Therapeutic Challenges

**DOI:** 10.32604/or.2026.089550

**Published:** 2026-10-28

**Authors:** Yutong Zhang, Xuanyi Lv, Zhihong Deng, Rui Zhuo, Jie Dong, Qiang Wang, Xuefeng Shi, Sijie Wu, Ulf D. Kahlert, Wenjie Shi, Yefang Hu, Li Xia

**Affiliations:** 1 Graduate School, Guangxi University of Chinese Medicine, Nanning, China; 2 Department of Breast Surgery, Guilin Municipal Hospital of Traditional Chinese Medicine, Guilin, China; 3 Molecular and Experimental Surgery, Clinic for General-, Visceral-, Vascular- and Transplantation Surgery, Medical Faculty and University Hospital Magdeburg, Otto-von-Guericke University, Magdeburg, Germany; 4 University Hospital for Gynecology, Pius Hospital, University Medicine Oldenburg, Oldenburg, Germany; 5 Department of Dermatology, Guilin People’s Hospital, Guilin, China

**Keywords:** Human epidermal growth factor receptor 2, breast cancer, therapeutic resistance, tumor microenvironment, cancer-associated fibroblasts

## Abstract

Human epidermal growth factor receptor 2 (HER2)-positive breast cancer is associated with aggressive disease and poor prognosis. Despite advances in HER2-targeted therapies, resistance remains a major clinical challenge. This review focuses on the mechanisms of resistance to HER2-targeted therapies, with particular emphasis on the tumor microenvironment (TME). The TME, composed of immune cells, cancer-associated fibroblasts (CAFs), and extracellular matrix components, significantly influences therapeutic responses. Immunosuppressive cells such as tumor-associated macrophages (TAMs), regulatory T cells (Tregs), and myeloid-derived suppressor cells (MDSCs) contribute to immune evasion and therapeutic resistance. Additionally, CAFs contribute to resistance by remodeling the extracellular matrix and creating physical barriers that restrict drug delivery and immune cell infiltration. Emerging therapeutic strategies targeting the TME components show promise in overcoming resistance and improving treatment outcomes. The development of predictive biomarkers, particularly those reflecting TME components, and the integration of spatial profiling technologies will be critical in optimizing personalized treatment strategies for HER2-positive breast cancer.

## 1 Introduction

Human epidermal growth factor receptor 2 (HER2) is amplified or overexpressed in approximately 15–20% of breast cancers, which is associated with aggressive clinical behavior and poor prognosis [1]. Despite advances in HER2-directed therapies, both *de novo* and acquired resistance remain major clinical obstacles [2,3]. Many patients with early-stage disease fail to achieve pathologic complete response after neoadjuvant therapy, and most with metastatic disease ultimately develop progression. Elucidating the mechanisms driving therapeutic escape therefore represents an urgent unmet need. Intrinsic resistance is present before treatment and is typically determined by pre-existing genomic, signaling, immune, or stromal features that limit the initial therapeutic response. In contrast, acquired resistance develops after an initial period of sensitivity and reflects treatment-driven clonal selection, adaptive signaling reprogramming, drug-tolerant persister states, immune escape, or remodeling of the tumor microenvironment (TME). Drug-tolerant persister (DTP) cells represent a reversible, non-mutational survival state characterized by slow proliferation, adaptive signaling, and epigenetic plasticity under therapeutic pressure. These cells may survive initial HER2-directed treatment and subsequently provide a reservoir for the emergence of stable acquired resistance [4].

Resistance to HER2-targeted therapy was historically attributed primarily to tumor cell-intrinsic alterations, including incomplete HER2 blockade, mutational activation of parallel signaling pathways, like PIK3CA mutations [5] or PTEN loss [6], emergence of truncated HER2 variants such as p95HER2 [7,8], and activation of alternative receptor tyrosine kinases [9]. However, a growing body of evidence has established that the TME plays a critical role in dictating therapeutic response and resistance. The TME is a complex ecosystem made up of non-malignant immune cells, such as Tregs, myeloid-derived suppressor cells (MDSCs), tumor-associated macrophages (TAMs), and effector lymphocytes, along with stromal cells like cancer-associated fibroblasts (CAFs), and a dynamic extracellular matrix (ECM). All of these components interact in a reciprocal crosstalk with tumor cells, shaping the biology of the disease. Fig. 1 illustrates a comprehensive overview of the TME components involved in resistance to HER2-targeted therapies, highlighting key players such as immune suppressive cells, immune checkpoints, dysregulated signaling pathways, and CAF-mediated barriers. Components of the TME actively remodel the signaling pathways, enforce immune suppression, and create physical and metabolic barriers, all of which contribute to resistance to HER2-targeted therapies [10,11].

Importantly, resistance mechanisms are not interchangeable across HER2-directed therapeutic modalities. Monoclonal antibodies such as trastuzumab and pertuzumab depend substantially on receptor accessibility, HER2-dependent signaling inhibition, and immune effector mechanisms, whereas HER2-directed tyrosine kinase inhibitors are more directly affected by intracellular pathway reactivation and adaptive signaling states. Antibody–drug conjugates, including T-DM1 and T-DXd, additionally depend on sufficient target accessibility, internalization, intracellular drug delivery, and payload sensitivity. Accordingly, throughout this review we distinguish, where supported by available evidence, between therapy-specific mechanisms and between intrinsic resistance, which is present before treatment, and acquired resistance emerging during therapeutic exposure.

A literature search was conducted in PubMed and Web of Science for studies published up to August 2026. Search terms included combinations of “HER2-positive breast cancer”, “HER2-targeted therapy”, “trastuzumab”, “pertuzumab”, “T-DM1”, “trastuzumab deruxtecan”, “tyrosine kinase inhibitor”, “drug resistance”, “tumor microenvironment”, “tumor-associated macrophages”, “regulatory T cells”, “myeloid-derived suppressor cells”, “cancer-associated fibroblasts”, “immune checkpoints”, and “spatial profiling”. Only English-language publications were considered. Original mechanistic studies, translational investigations, clinical studies, and major clinical trials directly relevant to resistance to HER2-targeted therapy were prioritized. Particular emphasis was placed on studies examining interactions between HER2-positive tumor cells and immune or stromal components of the tumor microenvironment. Recent high-quality reviews were additionally used to identify relevant primary studies. The present article was designed as a narrative review rather than a systematic review; therefore, no formal meta-analysis or PRISMA-based study-selection procedure was performed. Studies were selected based on their direct relevance to HER2-targeted therapy resistance, with priority given to mechanistic studies, translational investigations, clinical trials, and recent high-quality reviews addressing tumor–microenvironment interactions. Studies with limited relevance to HER2-positive breast cancer or without a clear link to therapeutic resistance were excluded. When findings were inconsistent across studies, greater weight was given to clinical and patient-derived evidence, while preclinical findings were used to provide mechanistic context. Conflicting results were described qualitatively and interpreted in light of differences in study design, model systems, treatment setting, and patient population.

**Figure 1:**
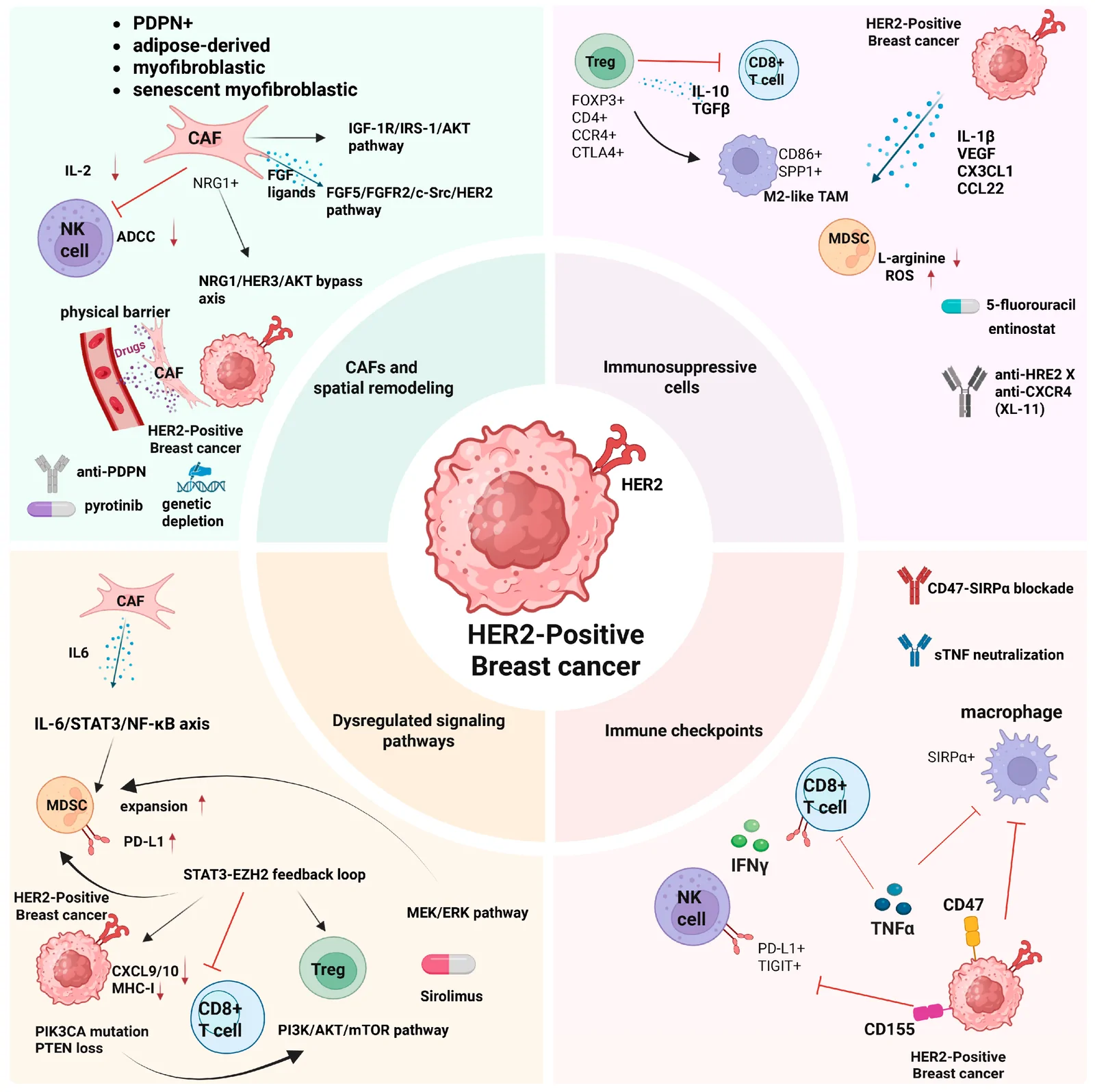
**Tumor microenvironment-mediated mechanisms of resistance in HER2-positive breast cancer.** The figure summarizes major immunosuppressive cell populations, immune-checkpoint pathways, tumor–microenvironment signaling interactions, and CAF-associated spatial mechanisms implicated in resistance to HER2-directed therapy. TAMs, Tregs, and MDSCs contribute to immune suppression through cytokine signaling, metabolic restriction, and inhibitory receptor pathways, whereas CAFs promote resistance through paracrine signaling, extracellular matrix remodeling, and spatial exclusion of immune effector cells. Tumor-cell-intrinsic signaling pathways are shown where they interact with or are reinforced by microenvironment-derived signals. Solid arrows indicate mechanisms supported by clinical or patient-derived evidence, whereas dashed arrows indicate mechanisms supported predominantly by preclinical studies. This distinction reflects the general maturity of the available evidence and does not constitute a formal evidence-grading system (Fig. 1 was created by the authors using BioRender.com). Note: ADCC, antibody-dependent cellular cytotoxicity; AKT, protein kinase B; CAF, cancer-associated fibroblast; CCL22, C-C motif chemokine ligand 22; CCR4, C-C chemokine receptor type 4; CD, cluster of differentiation; CTLA-4, cytotoxic T-lymphocyte-associated protein 4; CX3CL1, C-X3-C motif chemokine ligand 1; CXCL9/10, C-X-C motif chemokine ligands 9 and 10; EZH2, enhancer of zeste homolog 2; FGF5, fibroblast growth factor 5; FGFR2, fibroblast growth factor receptor 2; FOXP3, forkhead box P3; HER2, human epidermal growth factor receptor 2; HER3, human epidermal growth factor receptor 3; IFNγ, interferon gamma; IGF-1, insulin-like growth factor 1; IL, interleukin; IRS-1, insulin receptor substrate 1; MDSC, myeloid-derived suppressor cell; MEK, mitogen-activated protein kinase kinase; MHC-I, major histocompatibility complex class I; mTOR, mechanistic target of rapamycin; NF-κB, nuclear factor kappa B; NK, natural killer; NRG1, neuregulin 1; PD-L1, programmed death-ligand 1; PDPN, podoplanin; PI3K, phosphoinositide 3-kinase; PIK3CA, phosphatidylinositol-4,5-bisphosphate 3-kinase catalytic subunit alpha; PTEN, phosphatase and tensin homolog; ROS, reactive oxygen species; SIRPα, signal regulatory protein alpha; SPP1, secreted phosphoprotein 1; STAT3, signal transducer and activator of transcription 3; TAM, tumor-associated macrophage; TGFβ, transforming growth factor beta; TIGIT, T-cell immunoreceptor with immunoglobulin and ITIM domains; TNFα, tumor necrosis factor alpha; Treg, regulatory T cell; VEGF, vascular endothelial growth factor; ERK, extracellular signal-regulated kinase.

For conceptual clarity, resistance mechanisms discussed in this review are organized according to their relationship with the tumor microenvironment. Pre-existing microenvironmental resistance refers to immune suppression, stromal organization, or extracellular matrix features that are already present before therapy and reduce treatment sensitivity. Therapy-induced remodeling describes adaptive changes in immune or stromal populations that emerge during treatment. Tumor–microenvironment feedback refers to bidirectional signaling in which tumor-cell-intrinsic pathways are sustained or amplified by cytokines, growth factors, or stromal-derived signals. Immune escape encompasses suppression or dysfunction of T cells, NK cells, macrophages, and other effector populations, whereas spatial resistance refers to physical or architectural features that restrict immune-cell access, antibody binding, or drug penetration. Tumor-cell-intrinsic pathways are discussed only where they intersect with one or more of these microenvironmental processes. The relative contribution of these mechanisms is likely to vary across patients, disease sites, and treatment stages, with pre-existing immune or stromal states being particularly relevant to early or intrinsic resistance and therapy-induced remodeling becoming increasingly important during acquired resistance.

This review focuses on recent advances in understanding how the TME contributes to therapeutic resistance in HER2-positive breast cancer, with an emphasis on immunosuppressive cell populations, emerging immune checkpoints, dysregulated signaling pathways, adaptive CAF reprogramming, and CAF-mediated spatial barriers. By synthesizing preclinical discoveries and clinical observations, we aim to delineate the multifaceted mechanisms by which the TME orchestrates resistance and to highlight novel therapeutic vulnerabilities that may inform the next generation of combination strategies for this challenging disease (Table 1).

**Table 1:** Therapy-specific mechanisms of intrinsic and acquired resistance to HER2-directed treatment and their supporting evidence.

Therapeutic Modality	Representative Agents	Major Resistance Mechanisms Relevant to This Review	Predominant Resistance Type	Main Biological Context	Supporting Evidence	Ref.
HER2-directed TKIs	Lapatinib, neratinib, tucatinib, pyrotinib	Adaptive pathway rewiring; drug-tolerant persister states; ER–SGK3-dependent, AKT-independent mTORC1 activation; MAPK and other compensatory signaling	Predominantly acquired	Mainly tumor intrinsic, with potential TME reinforcement	Mainly preclinical evidence	[3,4]
Antibody–drug conjugates	T-DM1	Heterogeneous HER2 accessibility and microenvironment-supported survival signaling; activity may additionally be affected by pathway activation and spatial barriers	Intrinsic and acquired/context dependent	Tumor intrinsic + spatial/TME factors	Clinical and translational evidence for treatment efficacy; resistance mechanisms mainly preclinical/translational	[3,10]
HER2-directed monoclonal antibodies	Trastuzumab	Pre-existing PI3K/AKT/mTOR activation through PIK3CA mutation or PTEN loss; reduced immune-mediated activity caused by TAMs, Tregs and MDSCs; MUC4-mediated HER2 masking; CAF-mediated impairment of ADCC and antibody accessibility; IL-6/JAK/STAT3 and alternative RTK signaling	Intrinsic and acquired	Tumor intrinsic + TME crosstalk	Preclinical, translational, and clinical evidence	[5,6,10]
Antibody–drug conjugates	T-DXd	Heterogeneous HER2 expression and target accessibility; restricted tissue penetration in CAF-rich/ECM-rich tumors; spatial heterogeneity; dependence on sufficient HER2-mediated uptake and payload sensitivity despite the bystander effect	Intrinsic and acquired/context dependent	Tumor intrinsic + spatial/TME factors	Clinical efficacy established; resistance mechanisms mainly translational/preclinical	[8,10]
HER2-directed monoclonal antibodies	Pertuzumab, usually combined with trastuzumab	Paracrine HER3 reactivation, particularly through CAF-derived NRG1; stromal and spatial immune exclusion; persistence of downstream survival signaling despite dual HER2 blockade	Predominantly acquired or adaptive	TME–tumor crosstalk	Mainly preclinical and translational evidence	[9,10,11]

Note: HER2, human epidermal growth factor receptor 2; PI3K, phosphoinositide 3-kinase; AKT, protein kinase B; mTOR, mechanistic target of rapamycin; PIK3CA, phosphatidylinositol-4,5-bisphosphate 3-kinase catalytic subunit alpha; PTEN, phosphatase and tensin homolog; TAMs, tumor-associated macrophages; Tregs, regulatory T cells; MDSCs, myeloid-derived suppressor cells; MUC4, mucin 4; CAFs, cancer-associated fibroblasts; ADCC, antibody-dependent cellular cytotoxicity; IL-6, interleukin-6; JAK, Janus kinase; STAT3, signal transducer and activator of transcription 3; RTK, receptor tyrosine kinase; NRG1, neuregulin 1; TME, tumor microenvironment; TKI, tyrosine kinase inhibitor; ER, estrogen receptor; SGK3, serum/glucocorticoid-regulated kinase 3; MAPK, mitogen-activated protein kinase; T-DM1, trastuzumab emtansine; T-DXd, trastuzumab deruxtecan; ECM, extracellular matrix; mTORC, Mechanistic target of rapamycin complex 1.

## 2 Immunosuppressive Cells

The HER2-positive breast cancer TME contains diverse immunosuppressive cell populations that may contribute to both intrinsic and acquired resistance. Pre-existing enrichment of Tregs, MDSCs, or immunosuppressive TAMs can limit the immune-mediated activity of HER2-targeted antibodies, whereas prolonged treatment may promote adaptive immune escape through checkpoint upregulation, inflammatory signaling, macrophage reprogramming, and progressive T-cell or NK-cell dysfunction [12,13,14]. Among these, TAMs [15], regulatory T cells (Tregs) [16], and MDSCs represent three major axes of immune evasion that collectively undermine the efficacy of HER2-directed therapies.

### 2.1 Tumor-Associated Macrophages

TAMs are a prominent infiltrating immune population within the HER2-positive breast cancer TME and exhibit remarkable phenotypic plasticity. While classically activated M1-like macrophages possess antitumorigenic properties, the TME preferentially polarizes TAMs toward an immunosuppressive M2-like phenotype that promotes tumor progression and therapy resistance [17].

Recent single-cell transcriptomic profiling has identified a distinct SPP1+ macrophage subset enriched in HER2-positive tumors, displaying an M2-like immunosuppressive signature. These macrophages enhance Treg immunosuppressive function via CD86-CTLA4 signaling, promoting immune evasion and correlating with poor survival. Spatial transcriptomics and multiplex immunohistochemistry further confirmed the colocalization of SPP1+ macrophages and FOXP3+ Tregs within the HER2+ TME, highlighting their functional interplay as a key driver of tumor progression [13]. In addition, in HR+ breast cancer with high tumor-infiltrating lymphocyte (TIL) levels, SPP1+ macrophages also exhibit similar immunosuppressive interactions, suggesting a conserved role across breast cancer subtypes [18]. Furthermore, interleukin-8 (IL-8) secreted by TAMs undermines lapatinib efficacy in locally advanced HER2-positive breast cancer by activating the SRC/STAT3/ERK1/2 signaling pathway, which can be a compensatory bypass mechanism [19]. In addition, MMP11-expressing macrophages promote HER2-positive breast cancer-cell migration and monocyte recruitment through CCL2-CCR2-dependent MAPK signaling [20]. Moreover, TAM-derived soluble TNFα (sTNFα) promotes trastuzumab resistance by inducing mucin 4(MUC4)-mediated masking of the HER2 epitope, whereas sTNFα neutralization restores sensitivity by reprogramming macrophages toward an M1-like phenotype and enhancing NK-cell degranulation [21]. Collectively, TAMs contribute to HER2-targeted therapy resistance through macrophage-derived inflammatory cytokines, induction of tumor-cell bypass signaling, and MUC4-mediated masking of the HER2 epitope.

### 2.2 Regulatory T Cells

Tregs, characterized by FOXP3 expression, are a specialized subset of CD4+ T lymphocytes that tumors exploit to establish a profoundly immunosuppressive microenvironment [22]. Elevated Treg infiltration has been associated with progression, metastasis, and diminished therapeutic responses of HER2-positive breast cancer. Ohara et al. [23] analyzed 136 primary breast cancer specimens and found that FOXP3 mRNA levels were significantly elevated in HER2-positive tumors compared with normal breast tissue and were associated with increased IL-10 and TGF-β1 mRNA expression. These findings suggest an association between Treg infiltration and an immunosuppressive cytokine milieu, although they do not establish the cellular source of these cytokines or a direct causal role of Tregs in mediating immunosuppression.

Tregs do not operate in isolation but rather engage in intricate crosstalk with other immunosuppressive populations within the TME. As described above, the functional interplay between SPP1+ TAMs and Tregs via CD86-CTLA4 ligation exemplifies how distinct immunosuppressive compartments cooperate to reinforce immune evasion [13]. In addition, the CCL22-CCR4 chemokine axis can recruit Tregs in the HER2+ TME [24]. In HER2-positive tumors, elevated CCL22 levels create a gradient that recruits CCR4+ Tregs from the peripheral circulation into the tumor, prompting the development of therapies targeting Treg trafficking or selective depletion of tumor-infiltrating subsets [24].

### 2.3 Myeloid-Derived Suppressor Cells

MDSCs represent a population of immature myeloid-derived cells that expand pathologically in the TME and potently suppress antitumor immunity. The presence of MDSCs is associated with worse clinical outcomes, as their accumulation is linked to diminished responses to HER2-targeted therapies. A study of elderly patients with breast cancer showed that those with circulating HER2-reactive CD8^+^ T cells and a lower MDSC frequency had a 100% five-year survival rate, compared to just 38% in patients with higher MDSC levels and no HER2-reactive CD8^+^ T cells. This survival advantage was even observed in non-metastatic patients, underscoring the importance of the MDSC-CD8^+^ T cell axis in prognosis [25].

MDSCs contribute to therapeutic resistance through multiple mechanisms, including depletion of L-arginine and production of reactive oxygen species (ROS). The immunosuppressive function of MDSCs is further sustained by tumor-derived factors such as IL-1β, VEGF, and CX3CL1, which regulate their activation and recruitment [12]. Preclinical studies have suggested that 5-fluorouracil may reduce MDSC-mediated immunosuppression and enhance antitumor immune activity. In addition, epigenetic reprogramming has emerged as a promising strategy to attenuate MDSC-driven immunosuppression. A single-cell RNA sequencing study on HER2-overexpressing breast tumors showed that the histone deacetylase inhibitor entinostat shifts the TME from protumor to antitumor, primarily by altering myeloid cells. In entinostat-treated tumors, MDSCs adopted a less suppressive granulocytic phenotype, with altered NF-κB and STAT3 signaling, reducing their ability to inhibit T cell-mediated immunity [26]. All of these findings suggest that MDSCs contribute significantly to immune suppression within the HER2-positive breast cancer microenvironment and are closely associated with poor prognosis and therapeutic resistance.

While immunosuppressive cells within the tumor microenvironment (TME) play a critical role in promoting resistance to HER2-targeted therapies, another crucial aspect of immune evasion involves the upregulation of immune checkpoint molecules on tumor-infiltrating lymphocytes and other immune cells. This coordinated immune escape further complicates therapeutic efficacy and warrants attention in the development of combination strategies. Although TAMs, Tregs, and MDSCs are discussed separately, they should be considered components of an interconnected immunosuppressive network rather than independent resistance mechanisms. TAM-derived cytokines and costimulatory signals can promote Treg recruitment and function, while MDSC-derived metabolites and inflammatory mediators suppress effector T-cell and NK-cell activity and may reinforce macrophage polarization. The interaction between SPP1-positive macrophages and FOXP3-positive Tregs through CD86–CTLA4 signaling illustrates how distinct suppressive populations can cooperate within spatially defined tumor niches. These interactions may evolve during therapy, resulting in progressive immune dysfunction and reinforcing resistance to HER2-directed treatment.

### 2.4 ADCC and NK-Cell Dysfunction

Antibody-dependent cellular cytotoxicity (ADCC) is an Fc receptor-dependent immune effector mechanism in which antibody-coated tumor cells are recognized and killed predominantly by NK cells and represents an important component of trastuzumab activity. Within the HER2-positive TME, NK-cell activity can be impaired by multiple convergent mechanisms, including inhibitory checkpoint signaling, suppressive stromal interactions, metabolic restriction, and extracellular matrix-mediated spatial exclusion. These processes can reduce NK-cell activation and cytotoxicity and thereby attenuate trastuzumab-mediated ADCC. Among these microenvironmental mechanisms, CAF-mediated stromal and metabolic alterations represent an important source of NK-cell dysfunction. PDPN-positive CAFs can generate a metabolically unfavorable microenvironment that impairs NK-cell function and reduces ADCC, whereas senescent myofibroblastic CAFs can suppress NK-cell-mediated cytotoxicity through specialized extracellular matrix production. Together, these findings indicate that impaired ADCC represents an important interface between immune dysfunction, stromal remodeling, and resistance to HER2-directed antibodies.

## 3 Immune Checkpoint Upregulation

Resistance to HER2-directed therapy is mechanistically reinforced by the upregulation of immune checkpoint molecules on tumor-infiltrating lymphocytes and TAMs, which collectively drive T-cell exhaustion and innate immune escape [27].

In HER2-positive breast cancer, tumor-infiltrating CD8^+^ T cells and NK cells exhibit coordinated upregulation of multiple immune checkpoint receptors. A recent study showed that multiple inhibitory checkpoint receptors are co-expressed on intratumoral CD8^+^ T cells and NK cells in early-stage breast cancer, indicating a deeply exhausted immune phenotype within the TME. This pattern also suggests that single-agent checkpoint blockade may have limited efficacy because antitumor lymphocytes remain constrained by parallel inhibitory pathways [28]. In addition, PD-L1 upregulation is a characteristic feature of acquired trastuzumab resistance and appears to be driven, at least in part, by therapy-induced IFNγ signaling. By reinforcing an immunosuppressive microenvironment, this adaptive response may limit the durability of HER2-targeted treatment [14]. In addition to escaping T cell- and NK cell-mediated immunity, HER2-positive breast cancer cells may evade innate immune attack through the CD47-SIRPα checkpoint, which inhibits macrophage-mediated phagocytosis. This also suggests that CD47-targeted therapy could provide a complementary strategy to overcome trastuzumab resistance by activating an alternative antitumor effector pathway [29]. Furthermore, TIGIT-associated inhibitory signaling has been implicated in impaired antitumor lymphocyte function. High TIGIT co-expression has been observed on tumor-infiltrating lymphocytes in breast cancer [28], while experimental studies have shown that blockade of TIGIT-associated signaling can enhance human NK-cell function. Although TIGIT-associated inhibitory signaling has been reported in breast cancer and preclinical models, its specific role in HER2-positive breast cancer resistance remains unclear. Inflammatory mediators derived from the myeloid compartment may further reinforce checkpoint expression and lymphocyte dysfunction. Overall, these findings highlight immune checkpoint upregulation as a key feature of the immunosuppressive landscape in HER2-positive breast cancer and a potential contributor to resistance to HER2-directed therapy.

## 4 Tumor–Microenvironment Signaling Crosstalk and Adaptive Bypass Pathways

Tumor-cell-intrinsic signaling pathways can contribute directly to resistance to HER2-directed therapy; however, their relevance to the present review lies primarily in their bidirectional interaction with the tumor microenvironment. Cytokines, growth factors, immune-derived mediators, and stromal signals can sustain or reactivate intracellular survival pathways despite HER2 blockade, while tumor-cell signaling can in turn reshape immune-cell recruitment, checkpoint expression, stromal activation, and spatial organization. This section therefore focuses on signaling pathways at the tumor–microenvironment interface rather than on tumor-cell-intrinsic resistance mechanisms in isolation [30,31].

### 4.1 IL-6/STAT3/NF-κB Axis

IL-6/STAT3 signaling may contribute to both intrinsic and acquired resistance, although through different biological contexts. In the intrinsic setting, constitutive STAT3 activation or a pre-existing IL-6-rich microenvironment may reduce the initial response to trastuzumab [32]. During acquired resistance, prolonged HER2 blockade can amplify inflammatory feedback signaling, promote the expansion of stem-like tumor-cell populations, and reinforce STAT3-dependent survival programs [30]. The IL-6/STAT3/NF-κB network operates across multiple cellular compartments within the HER2-positive TME. IL-6 may be produced by tumor cells, TAMs, MDSCs, and CAFs, whereas its downstream effects depend on the responding cell type. In tumor cells, IL-6-mediated JAK/STAT3 activation promotes survival, stem-like properties, and resistance to HER2 blockade. In immune cells, STAT3 and NF-κB signaling can reinforce immunosuppressive polarization, MDSC expansion, and impaired antitumor immunity [26,30]. In PTEN-deficient tumor models, persistent HER2 blockade can activate an IL-6-driven inflammatory feedback loop involving STAT3 and NF-κB, which promotes tumor-cell survival, MDSC expansion, and PD-L1 upregulation, thereby reinforcing therapeutic resistance [30]. Notably, this signaling axis sustains cancer cell survival and expands the cancer stem cell population, which displays an EMT phenotype and secretes over 100-fold more IL-6 than parental cells, perpetuating a feed-forward inflammatory cascade [30]. Clinically and experimentally, increased STAT3 activation has been associated with trastuzumab resistance and poor therapeutic response in HER2-positive breast cancer. Stromal IL-6 derived from CAFs can further reinforce this pathway through paracrine signaling, as discussed in the CAF section [31,32]. Importantly, interrupting this loop with an IL-6 receptor antibody effectively reduces the cancer stem cell compartment, restores trastuzumab sensitivity, and suppresses tumor growth and metastasis in preclinical models [30]. These findings also highlight the contribution of stem-like tumor-cell states to acquired resistance. CSC plasticity may be reinforced by inflammatory and stromal signals within the TME, linking tumor-cell adaptation with microenvironmental remodeling. Although additional pathways such as Wnt/β-catenin, Notch, and Hedgehog have been implicated in breast cancer stemness, their specific contribution to HER2-directed resistance remains less well defined and is therefore not discussed in detail here.

### 4.2 EZH2-Mediated Epigenetic Immune Exclusion

Enhancer of zeste homolog 2 (EZH2), the catalytic subunit of polycomb repressive complex 2, can promote immune evasion by establishing a transcriptionally repressive tumor state. EZH2-mediated H3K27 trimethylation suppresses T-cell-recruiting chemokines, including CXCL9 and CXCL10, and can reduce expression of components of the MHC class I antigen-presentation machinery. These changes impair CD8^+^ T-cell recruitment and tumor-cell recognition, thereby favoring an immune-excluded microenvironment [33,34]. EZH2 may also reinforce immunosuppression through effects on non-malignant immune populations. Its activity has been linked to maintenance of FOXP3-dependent regulatory T-cell programs and to the persistence of myeloid-derived suppressor cells, although these effects are context- and cell-type-dependent [35,36,37,38]. Accordingly, elevated EZH2 activity may contribute to an immunologically cold TME characterized by reduced effector-cell infiltration and impaired antitumor immunity.

In HER2-positive breast cancer models, increased H3K27 trimethylation was associated with suppression of interferon-responsive programs and reduced sensitivity to HER2-directed antibodies. Pharmacological reduction of H3K27me3 restored interferon-related antitumor activity and enhanced the efficacy of HER2-targeted treatment [39]. These findings support EZH2-mediated epigenetic repression as a potential link between tumor-cell chromatin regulation and TME-dependent immune resistance. However, the predictive value of EZH2 activity and the therapeutic benefit of combining EZH2 inhibition with HER2-targeted therapy remain to be established clinically.

### 4.3 PI3K/AKT/mTOR Pathway Activation

Pre-existing PIK3CA mutations or PTEN loss can confer intrinsic resistance by maintaining downstream survival signaling despite HER2 blockade [5,6]. Acquired resistance may instead involve adaptive pathway rewiring, drug-tolerant persister states, or alternative downstream effectors that sustain mTORC1 signaling during treatment [4,40]. These findings underscore the clinical relevance of PI3K pathway hyperactivation, which is frequently driven by PIK3CA mutations or PTEN loss, as a key determinant of therapeutic resistance in HER2-positive disease.

Mechanistically, preclinical studies have directly established that PIK3CA hotspot mutations, such as E545K and H1047R, can drive resistance by constitutively activating the PI3K/AKT/mTOR pathway independent of HER2 receptor control. In PIK3CA-mutant HER2-positive breast cancer cells, persistent AKT phosphorylation is maintained despite HER2 blockade, confirming that the mutation itself sustains pathway activity [41,42]. Notably, pharmacological inhibition of PI3K or AKT effectively restores sensitivity to HER2-targeted agents, providing direct evidence that the pathway is the functional mediator of resistance. In addition to restoring tumor-cell sensitivity to HER2 blockade, selective inhibition of PI3K signaling may enhance the immune-mediated activity of anti-HER2 therapy. In preclinical HER2-positive breast cancer models, the PI3K p110α-selective inhibitor A66 enhanced the efficacy of anti-HER2/neu antibody treatment and exerted stronger immunostimulatory effects than pan-PI3K inhibition [43]. Recent work has identified an alternative mechanism of pathway reactivation in drug-tolerant persister cells. HER2 TKI exposure can induce a luminal-like persister population in which estrogen receptor-dependent SGK3 expression enables AKT-independent mTORC1 activation, allowing survival despite PI3K/AKT inhibition [4]. Collectively, PI3K/AKT/mTOR activation sustains tumor-cell survival during HER2 blockade and may also attenuate the immune-mediated activity of HER2-targeted therapy.

### 4.4 MEK/ERK Pathway

MAPK pathway reactivation represents an important bypass mechanism in HER2-positive breast cancer, arising through both genomic alterations and treatment-induced adaptive signaling rewiring that sustains tumor-cell survival despite HER2 blockade [40,44]. Although these mechanisms are primarily tumor-cell intrinsic, their relevance to TME-mediated resistance lies in the bidirectional crosstalk between tumor cells and the surrounding immune and stromal compartments. Microenvironment-derived cytokines and growth factors may sustain MEK/ERK signaling despite HER2 blockade, whereas persistent MAPK activation may in turn promote immune evasion by altering immune-checkpoint expression, myeloid-cell recruitment, and antigen-presentation pathways [45]. The precise contribution of this crosstalk in HER2-positive breast cancer remains incompletely defined; nevertheless, current evidence suggests that MEK/ERK activation may serve as an interface between tumor-cell adaptive resistance and microenvironmental immune suppression.

Adaptive STAT3 activation may also limit the efficacy of pathway-directed therapy. In oncogene-addicted cancer models, including HER2-amplified cells, MEK/ERK inhibition can trigger compensatory FGFR- and JAK-dependent STAT3 activation, thereby sustaining tumor-cell survival [46]. Although this feedback mechanism is primarily tumor-cell intrinsic, cytokines and growth factors within the TME may further reinforce STAT3 signaling. Its specific contribution to HER2-targeted therapy resistance in patients, however, remains incompletely defined. These signaling pathways should not be viewed as exclusively tumor-cell-intrinsic mechanisms. Their activity can be sustained or amplified by stromal-derived cytokines and growth factors, creating bidirectional feedback between tumor cells and the surrounding microenvironment. CAFs represent a major source of such signals and can reinforce IL-6/JAK/STAT3, PI3K/AKT, HER3, and Src-dependent survival pathways. The following section therefore examines CAF-mediated resistance as both a structural barrier and a source of adaptive paracrine signaling that connects stromal remodeling with tumor-cell survival.

## 5 Cancer-Associated Fibroblasts and Spatial Remodeling

CAF-mediated resistance comprises both pre-existing and therapy-associated components. Pre-existing CAF subsets and extracellular matrix organization may limit drug access or provide alternative survival signals, whereas therapy-associated CAF reprogramming can reshape the secretome and intensify stromal and spatial barriers during treatment [47].

### 5.1 Pro-Resistant Subsets of Cancer-Associated Fibroblasts

CAFs are functionally diverse, and pro-resistant extracellular matrix (ECM)-remodeling CAF populations are enriched in HER2-positive tumors compared with luminal subtypes. These CAFs contribute to trastuzumab resistance by shaping an immunosuppressive TME and reducing antibody efficacy [47].

Among the pro-resistant CAF subpopulations, PDPN+ CAFs have emerged as key drivers of trastuzumab resistance in HER2-positive breast cancer. Pu et al. [48]. demonstrated that PDPN+ CAFs are significantly enriched in trastuzumab-resistant tumors. These CAFs contribute to resistance by creating an immunosuppressive microenvironment that impairs NK-cell function and reduces trastuzumab-mediated ADCC. By secreting tryptophan-catabolizing enzymes, PDPN+ CAFs create a metabolically unfavorable microenvironment that impairs NK cell function, directly diminishing trastuzumab’s therapeutic efficacy. In preclinical murine models, anti-PDPN antibody treatment significantly enhanced antitumor efficacy and prolonged PFS when combined with trastuzumab. Apart from PDPN+ CAFs, Zhang et al. [49]. identified adipose-derived CAFs with elevated IGF-1 expression in resistant tumors, inducing trastuzumab resistance via IGF-1-mediated activation of the IGF-1R/IRS-1/AKT pathway in HER2-positive epithelial cells.

Furthermore, a subset of TGF-β-activated myofibroblastic CAFs (myCAFs) has been identified as specifically enriched in trastuzumab-resistant HER2-positive breast tumors. These TGF-β-responsive CAFs are characterized by a podoplanin-positive, immunomodulatory phenotype and are associated with low IL-2 activity within the TME, thereby impairing effective antitumor immunity [50]. Importantly, stromal-targeted activation of the IL-2 pathway in unresponsive tumors was found to restore trastuzumab sensitivity, demonstrating the potential for reversing this CAF-driven resistance mechanism [50]. More recently, a distinct population of senescent myofibroblastic CAFs (senCAFs) has been identified in HER2-positive breast cancer, which potently suppress NK cell-mediated cytotoxicity through the secretion of a specialized ECM, thereby limiting ADCC and promoting tumor progression [51]. Collectively, these findings underscore the multifaceted contributions of heterogeneous CAF subsets to therapeutic escape, reinforcing the concept that precise subset characterization rather than broad stromal ablation is essential for overcoming CAF-driven resistance in HER2-positive breast cancer.

### 5.2 Adaptive Reprogramming of CAFs and Bypass Pathway Activation

Anti-HER2 therapy can induce adaptive reprogramming of the surrounding stroma, thereby enabling CAFs to protect residual tumor cells through treatment-induced secretome remodeling. Multi-omics analyses have shown that combined treatment with trastuzumab, pertuzumab, and docetaxel alters the secretion of cytokines, proteins, and miRNAs by CAFs, creating a form of non-cell-autonomous resistance in which stromal cells compensate for HER2 blockade by supplying alternative survival signals [52].

Several CAF-derived factors can activate such bypass pathways. CAF-secreted IL-6 stimulates JAK/STAT3, PI3K/AKT, and NF-κB signaling in adjacent HER2-positive tumor cells, promoting survival, stem-like properties, and resistance to HER2-targeted therapy. Accordingly, IL-6 neutralization or inhibition of its downstream signaling pathways can partially restore trastuzumab sensitivity [31]. CAF-derived growth factors may also reactivate receptor tyrosine kinase signaling despite HER2 inhibition. Neuregulin-1 (NRG1) promotes HER3 phosphorylation and the formation of signaling-competent HER2–HER3 complexes, thereby restoring downstream PI3K/AKT signaling. Genetic depletion of CAF-derived NRG1 reverses resistance, whereas pertuzumab only partially suppresses this paracrine pathway, supporting the role of the NRG1/HER3/AKT axis in weakening dual HER2 blockade [53]. Similarly, tumor-associated fibroblast-derived FGF5 activates an FGFR2/c-Src/HER2 escape pathway, allowing HER2 signaling to persist in the presence of trastuzumab; this resistance can be attenuated by FGFR inhibition [54].

Together, these findings indicate that therapy-induced CAF reprogramming promotes resistance through a network of paracrine signals that reactivate JAK/STAT3, PI3K/AKT, and Src-dependent survival pathways despite continued HER2 blockade.

### 5.3 CAF-Mediated Spatial Barrier and Immune Exclusion

Beyond paracrine signaling, CAFs can form physical and spatial barriers that restrict drug penetration and exclude immune effector cells from tumor nests. CAF-derived hyaluronan and other extracellular matrix components interact with CD44 on tumor cells, creating steric hindrance that masks the trastuzumab-binding epitope on HER2 and promotes caveolin-1-dependent HER2 internalization, thereby reducing antibody accessibility [55,56]. Spatial analyses further support a role for CAF organization in immune exclusion. In tumors resistant to docetaxel, carboplatin, trastuzumab, and pertuzumab, dense CAF barriers limited CD8^+^ T-cell access to tumor nests, while proliferating Ki67^+^ tumor cells were located in close proximity to CAF-rich regions. Similarly, myofibroblastic and ECM-remodeling CAFs were enriched in TGF-β-active stromal regions associated with CD8^+^ T-cell exclusion [57,58]. In contrast, EMILIN1-expressing inflammatory CAFs were associated with greater CD8^+^ T-cell infiltration and more favorable clinical outcomes [58]. These findings indicate that the spatial distribution and functional heterogeneity of CAF subsets can influence both antibody accessibility and immune-cell infiltration in HER2-positive breast cancer.

## 6 Biomarkers and Patient Stratification

Given that TME-mediated resistance is driven by interacting immune, stromal, and spatial mechanisms, single biomarkers are unlikely to provide sufficient predictive value. Importantly, prognostic biomarkers, which are associated with clinical outcome independent of a specific treatment, should be distinguished from predictive biomarkers that identify differential benefit from a particular therapeutic intervention. This distinction is especially relevant for TME-derived biomarkers, as many immune and stromal features associated with poor outcome have not yet been prospectively validated for treatment selection. Instead, patient stratification may require integrated assessment of immune-cell composition, checkpoint activity, CAF phenotypes, extracellular matrix organization, and their spatial relationships with HER2-positive tumor cells. Immune-enriched biomarkers may include the abundance and functional state of Tregs, MDSCs, and immunosuppressive macrophage subsets. For example, enrichment of SPP1^+^ macrophages together with FOXP3^+^ Tregs identifies a highly immunosuppressive microenvironment associated with adverse outcomes [13]. At present, this feature should therefore be considered primarily prognostic and biologically informative rather than a prospectively validated predictor of response to HER2-directed therapy. Likewise, elevated MDSC burden and reduced HER2-reactive CD8^+^ T-cell responses may indicate impaired antitumor immunity and have mainly prognostic or immune-state relevance [25]. Adaptive checkpoint expression, including PD-L1 and TIGIT-associated inhibitory signaling, may further identify tumors in which lymphocyte dysfunction limits the immune-mediated activity of HER2-targeted antibodies [14,28,59]. However, the predictive value of these markers remains context-dependent, and their use for treatment selection requires further prospective validation.

Stromal biomarkers may be particularly relevant for identifying tumors dominated by CAF-mediated resistance. Enrichment of PDPN^+^ CAFs or TGF-β-responsive myofibroblastic CAFs has been associated with impaired antibody-dependent cellular cytotoxicity, immune suppression, and reduced trastuzumab responsiveness [48,50]. However, CAF abundance alone may be insufficient because distinct CAF subsets can exert opposing effects. Spatial organization may therefore provide additional predictive information. Dense CAF-rich regions and TGF-β-active stromal niches are associated with CD8^+^ T-cell exclusion, whereas EMILIN1-expressing inflammatory CAFs are linked to greater immune-cell infiltration and more favorable outcomes [57,58]. Similarly, hyaluronan-rich extracellular matrix and MUC4-mediated HER2 masking may indicate impaired antibody accessibility rather than loss of HER2 expression itself [21,55,56].

Spatial profiling technologies may provide an important tool for capturing resistance states that cannot be resolved by bulk molecular analysis alone. Platforms such as imaging mass cytometry, multiplexed ion beam imaging, and digital spatial profiling can simultaneously characterize tumor cells, immune populations, CAF subsets, checkpoint expression, and their spatial relationships within intact tissue. In HER2-positive breast cancer, spatial proteomic profiling has already shown that treatment-induced changes in tumor and immune organization may provide predictive information beyond baseline HER2 status alone [60]. However, the clinical application of these technologies remains limited by platform variability, tissue requirements, sampling bias, analytical complexity, and the absence of prospectively validated spatial thresholds for treatment selection.

## 7 Therapeutic Platforms for Overcoming TME-Mediated Resistance

Current therapeutic approaches for TME-mediated resistance include antibody-based immunomodulation, pathway-directed small molecules, and ADCs capable of partially overcoming spatial heterogeneity. These platforms differ in the microenvironmental barriers they address and in the extent to which their activity depends on immune-cell function, stromal organization, and target accessibility. From a clinical perspective, the optimal strategy may depend on the dominant resistance context rather than on HER2 status alone. Tumors with preserved HER2 expression but impaired immune effector function may be more suitable for approaches that restore or redirect antitumor immunity, including checkpoint modulation or bispecific antibodies. In contrast, tumors dominated by stromal-derived survival signaling may require pathway-directed combinations targeting PI3K/AKT/mTOR, JAK/STAT3, HER3, or related bypass pathways. ADCs may be particularly useful when HER2 remains sufficiently accessible but intratumoral heterogeneity limits the efficacy of conventional HER2 blockade, although dense stromal architecture and impaired target accessibility may still restrict drug delivery. These considerations support a resistance-adapted treatment framework in which immune competence, stromal organization, pathway activation, and spatial HER2 accessibility are assessed together rather than independently.

### 7.1 Antibody-Based Therapies

#### 7.1.1 Monoclonal Antibodies

Preclinical studies support several antibody-based strategies for reversing TME-mediated resistance. CD47-SIRPα blockade enhances trastuzumab-mediated macrophage phagocytosis, whereas inhibition of TIGIT-associated signaling may improve NK-cell activity during antibody-dependent cellular cytotoxicity [29,59]. Selective neutralization of soluble TNFα and targeting of PDPN^+^ CAFs have also restored or enhanced trastuzumab activity in preclinical models by alleviating myeloid- and stromal-mediated resistance, respectively [21,48]. Clinical experience with PD-1/PD-L1 blockade in HER2-positive breast cancer has produced limited and context-dependent efficacy. In the phase Ib/II PANACEA trial, patients had trastuzumab-resistant advanced HER2-positive breast cancer and were prospectively stratified according to PD-L1 status. Among 40 patients with PD-L1-positive tumors treated with pembrolizumab plus trastuzumab, six achieved an objective response (15%), whereas no objective responses were observed among 12 patients with PD-L1-negative tumors. Treatment-related grade 3–5 adverse events occurred in 29% of patients, underscoring the need to balance immune-mediated efficacy with toxicity [61].

In the randomized phase II KATE2 trial, patients with previously treated HER2-positive advanced breast cancer received T-DM1 with either atezolizumab or placebo. Median progression-free survival was 8.2 months with atezolizumab versus 6.8 months with placebo (HR 0.82, 95% CI 0.55–1.23; *p* = 0.33), demonstrating no significant benefit in the overall population. Serious adverse events occurred more frequently with atezolizumab than placebo (33% versus 19%). Exploratory analyses suggested that PD-L1-positive tumors might represent a more responsive subgroup, but this observation remains hypothesis-generating [62].

Similarly, the phase III IMpassion050 trial did not demonstrate improved pathological complete response with the addition of atezolizumab to neoadjuvant chemotherapy plus trastuzumab and pertuzumab. Pathological complete response rates were 62.4% with atezolizumab and 62.7% with placebo in the intention-to-treat population. In the PD-L1-positive subgroup, the corresponding rates were 64.2% and 72.5%, respectively. Grade 3–4 and serious adverse events were more frequent in the atezolizumab group, and five grade 5 adverse events occurred among patients assigned to atezolizumab [63]. Collectively, these trials demonstrate that checkpoint inhibition cannot currently be considered a broadly effective strategy for HER2-positive breast cancer. PD-L1 status alone appears insufficient for patient selection, supporting the need for composite biomarkers incorporating immune infiltration, spatial organization, myeloid suppression, and additional checkpoint pathways.

#### 7.1.2 Bispecific Antibodies

Bispecific antibodies provide a spatially directed strategy by linking HER2-positive tumor cells to immune effector or regulatory pathways. HER2×CD3 T-cell engagers, including M802, induce HER2-dependent T-cell activation and tumor-cell cytotoxicity, whereas optimization of HER2- and CD3-binding affinities may improve the balance between efficacy and on-target/off-tumor toxicity [64,65]. Other formats address specific immunosuppressive contexts. The HER2×CCR4 bispecific antibody XL-11 promotes tumor-localized depletion of CCR4-positive Tregs in preclinical models [66]. Early clinical evidence has also been reported for the combination of the HER2-directed bispecific antibody KN026 with the PD-L1/CTLA-4 bispecific antibody KN046, which showed antitumor activity and immune-cell activation in previously treated HER2-positive metastatic breast cancer [67]. However, the suitability of these platforms may depend on the TME context. HER2×CD3 antibodies are likely to require sufficient T-cell infiltration and functional competence, whereas HER2×CCR4 approaches may be more relevant to Treg-enriched tumors. Immune exclusion, T-cell exhaustion, antigen heterogeneity, and cytokine-related toxicity remain important barriers, supporting integrated assessment of HER2 density and immune composition when selecting bispecific strategies.

#### 7.1.3 Small-Molecule and Pathway-Directed Therapies

Small-molecule therapies may counteract TME-supported resistance by inhibiting signaling pathways sustained by stromal and immune-cell-derived cues. Clinical development has focused mainly on the PI3K/AKT/mTOR pathway. Alpelisib combined with T-DM1 showed clinical activity in trastuzumab-pretreated HER2-positive metastatic breast cancer, although the small, non-randomized study population and treatment-related toxicity limit definitive conclusions [68]. Sirolimus-based combinations have similarly shown encouraging activity in selected patients with PI3K/AKT/mTOR pathway activation [69]. The clinical effectiveness of pathway-directed therapy may be limited by compensatory signaling and systemic toxicity. Inhibition of a single pathway node can be bypassed through HER3, MEK/ERK, JAK/STAT3, or other survival programs, supporting the use of these agents in biomarker-selected combination strategies rather than as broadly applicable resistance-reversing treatments [40,44,46,53]. Some small molecules may also reshape the TME indirectly. Entinostat can attenuate myeloid-cell-mediated immunosuppression, sepiapterin has been explored for macrophage metabolic reprogramming, and pyrotinib may disrupt fibroblast-associated spatial barriers and improve CD8^+^ T-cell access in resistant tumors [26,57,70]. These observations suggest that small-molecule therapies may be most effective when they simultaneously suppress tumor-cell signaling and weaken microenvironmental support.

#### 7.1.4 Taxanes and Emerging Taxane Derivatives

Taxanes remain important components of chemotherapy-containing regimens for HER2-positive breast cancer and may influence the efficacy of HER2-directed treatment through their direct cytotoxic effects and their interactions with the tumor microenvironment. However, resistance to taxanes is mechanistically distinct from resistance to HER2-targeted agents and includes altered drug transport, microtubule dynamics, metabolic disposition, and multidrug-resistance pathways. Newer taxane derivatives have been developed in an effort to improve pharmacological properties and activity in drug-resistant tumors. Felotaxel (SHR110008), a semisynthetic docetaxel derivative, has shown preclinical antitumor activity and has been investigated with respect to pharmacokinetics, tissue distribution, excretion, and plasma protein binding. In rats, felotaxel demonstrated rapid tissue distribution, with tissue concentrations exceeding plasma concentrations in most examined organs, whereas renal exposure was particularly high. Additional LC–MS/MS-based pharmacokinetic studies established analytical approaches for quantifying felotaxel in biological matrices. These investigations provide useful pharmacological information for the development of novel taxane-based combinations; however, direct evidence linking felotaxel to HER2-specific or TME-mediated resistance in breast cancer is currently limited. Consequently, its potential role in overcoming resistance to HER2-directed therapy remains to be established [71,72].

### 7.2 Antibody Drug Conjugates

Antibody–drug conjugates are not directly TME-targeted, but their activity is closely linked to intratumoral spatial organization. T-DXd contains a cleavable linker and a membrane-permeable topoisomerase I inhibitor payload, enabling bystander killing of neighboring cells after HER2-dependent internalization and payload release. This property may partially compensate for heterogeneous HER2 expression, although sufficient target-mediated uptake remains necessary for effective drug delivery. Resistance to HER2-directed ADCs involves both tumor-cell-intrinsic and microenvironmental mechanisms. For T-DM1, reduced HER2 expression, impaired internalization and lysosomal processing, and altered intracellular payload handling may decrease cytotoxic efficacy. T-DXd may partially overcome HER2 heterogeneity through its cleavable linker and membrane-permeable topoisomerase I inhibitor payload, which enables bystander killing after HER2-dependent internalization [73]. Nevertheless, reduced HER2 accessibility, altered payload sensitivity, and changes in DNA-damage response may still contribute to resistance. Importantly, CAF-rich extracellular matrix, limited tissue penetration, and spatially heterogeneous target accessibility may further restrict ADC delivery. Thus, resistance to T-DM1 and T-DXd reflects an interaction between intrinsic ADC-processing mechanisms and microenvironmental barriers.

In DESTINY-Breast03, T-DXd significantly improved progression-free and overall survival compared with T-DM1 in previously treated HER2-positive metastatic breast cancer [74]. Despite this substantial clinical benefit, T-DXd is associated with a clinically relevant risk of interstitial lung disease (ILD)/pneumonitis, highlighting the need for careful monitoring, early recognition, and timely management during treatment. However, its efficacy may still be constrained by CAF-rich extracellular matrix, limited tissue penetration, heterogeneous target accessibility, and site-specific stromal architecture. Spatial proteomic analyses further suggest that treatment-induced changes in tumor and immune organization may provide predictive information beyond baseline HER2 status [60]. Therefore, evaluation of ADC responsiveness may require consideration of HER2 density and spatial heterogeneity together with stromal organization, metastatic site, and payload sensitivity. These therapeutic platforms differ in both their mode of action and their dependence on the surrounding microenvironment. Antibody-based approaches primarily rely on restoring or redirecting immune effector function, whereas small-molecule therapies are more suited to suppressing microenvironment-supported signaling networks. ADCs occupy an intermediate position, as they remain HER2-directed but may partially compensate for intratumoral heterogeneity through bystander payload activity. Despite these differences, all three platforms face common translational barriers, including incomplete target accessibility, adaptive pathway compensation, treatment-related toxicity, and marked spatial and temporal heterogeneity of the TME. Their successful clinical application will therefore depend on rational combination strategies and prospective validation of biomarkers that reflect both tumor-intrinsic and microenvironmental resistance states. The major TME-associated resistance contexts and corresponding therapeutic strategies are summarized in Table 2.

**Table 2:** Tumor microenvironment-mediated resistance mechanisms and corresponding therapeutic approaches in HER2-positive breast cancer.

Suppressive Components	Representative Markers	Contribution	Therapeutic Approaches	Refs.
TAMs	SPP1^+^ TAMs; IL-8; sTNFα;	Immune suppression, bypass signaling, and HER2 masking	sTNFα neutralization; macrophage reprogramming	[13,19,21,70]
Tregs	FOXP3; CD86-CTLA-4; CCL22-CCR4	Suppression of effector T-cell responses and reinforcement of an immunosuppressive niche	HER2×CCR4 bispecific antibodies	[13,23,24,66]
MDSCs	ROS; arginine depletion; STAT3/NF-κB	Inhibition of T-cell function and attenuation of antibody-mediated immunity	Entinostat-mediated myeloid reprogramming	[12,25,26]
Immune checkpoints	PD-1/PD-L1; TIGIT; CD47-SIRPα; parallel inhibitory receptors	T-cell, NK-cell, and macrophage dysfunction during HER2-directed therapy	PD-1/PD-L1 blockade; investigational TIGIT-directed strategies; checkpoint bispecifics	[14,28,29,63,67]
TME-supported signaling and epigenetic immune exclusion	IL-6/JAK/STAT3/NF-κB; PI3K/AKT/mTOR; MEK/ERK; EZH2-H3K27me3	Sustained tumor-cell survival, adaptive pathway activation, and epigenetic immune exclusion	Pathway-directed combinations; EZH2 inhibition	[30,39,46,68,69]
Pro-resistant CAF subsets	PDPN+ CAFs; TGF-β-responsive myCAFs; senCAFs	Impaired ADCC, NK-cell suppression, and stromal immunosuppression	Anti-PDPN therapy; CAF-subset-directed or stromal immunotherapy	[48,50,51]
Adaptive CAF reprogramming and paracrine bypass	IL-6; NRG1; FGF5; IGF-1	Reactivation of JAK/STAT3, HER3/PI3K/AKT, and FGFR2/Src signaling despite HER2 blockade	Inhibitors of IL-6/JAK/STAT3, NRG1/HER3, FGFR2, IGF-1R, or PI3K/AKT/mTOR signaling	[31,49,52,68,69]
CAF-mediated spatial barriers	Hyaluronan/CD44; dense ECM, CAF-mediated immune exclusion	Reduced antibody accessibility, impaired drug penetration, and restricted CD8^+^ T-cell infiltration	Stromal remodeling; ECM-directed strategies	[55,56,57,58]

Note: ADCC, antibody-dependent cellular cytotoxicity; AKT, protein kinase B; CAFs, cancer-associated fibroblasts; CCL22, C-C motif chemokine ligand 22; CCR4, C-C chemokine receptor type 4; CD, cluster of differentiation; CTLA-4, cytotoxic T-lymphocyte-associated protein 4; ECM, extracellular matrix; ERK, extracellular signal-regulated kinase; EZH2, enhancer of zeste homolog 2; FGF5, fibroblast growth factor 5; FGFR2, fibroblast growth factor receptor 2; FOXP3, forkhead box P3; H3K27me3, trimethylation of histone H3 at lysine 27; HER2, human epidermal growth factor receptor 2; HER3, human epidermal growth factor receptor 3; IGF-1, insulin-like growth factor 1; IGF-1R, insulin-like growth factor 1 receptor; IL-6, interleukin 6; JAK, Janus kinase; MDSCs, myeloid-derived suppressor cells; MEK, mitogen-activated protein kinase kinase; mTOR, mechanistic target of rapamycin; myCAFs, myofibroblastic cancer-associated fibroblasts; NF-κB, nuclear factor kappa B; NK, natural killer; NRG1, neuregulin 1; PD-1, programmed cell death protein 1; PD-L1, programmed death-ligand 1; PDPN, podoplanin; PI3K, phosphoinositide 3-kinase; ROS, reactive oxygen species; senCAFs, senescent cancer-associated fibroblasts; SIRPα, signal regulatory protein alpha; SPP1, secreted phosphoprotein 1; STAT3, signal transducer and activator of transcription 3; sTNFα, soluble tumor necrosis factor alpha; TAMs, tumor-associated macrophages; TGF-β, transforming growth factor beta; TIGIT, T-cell immunoreceptor with immunoglobulin and ITIM domains; TME, tumor microenvironment; Tregs, regulatory T cells.

## 8 Conclusions and Future Perspectives

Resistance to HER2-targeted therapy is not determined solely by tumor-cell-intrinsic alterations but also by dynamic interactions among malignant cells, immune populations, fibroblasts, and the extracellular matrix. These interactions can suppress antibody-dependent immune activity, sustain compensatory survival signaling, restrict drug penetration, and generate spatially protected tumor niches. The TME should therefore be considered an active determinant of both primary and acquired resistance rather than a passive background feature.

Distinguishing intrinsic from acquired resistance has important translational implications. Baseline molecular and microenvironmental profiling may help identify pre-existing immune suppression, stromal exclusion, or pathway activation, whereas acquired resistance is more likely to require serial assessment of treatment-induced changes. Because these features may differ between lesions and evolve during therapy, single pretreatment biopsies are unlikely to capture the full resistance landscape. Integration of on-treatment tissue analysis, liquid biopsy, and spatially resolved profiling may provide a more accurate view of emerging therapeutic escape.

Future progress will depend on moving beyond single-marker stratification toward composite biomarkers that integrate HER2 expression and accessibility with immune-cell state, checkpoint activity, CAF composition, extracellular matrix organization, and spatial cell–cell relationships. Prospective studies should incorporate longitudinal molecular and spatial profiling to determine whether changes in these features can identify emerging resistance before clinical progression. Biomarker-enriched trials should then test whether therapeutic combinations selected according to the dominant resistance context improve outcomes compared with treatment selection based primarily on HER2 status. Immune-directed combinations may be prioritized in tumors with preserved HER2 expression but impaired immune effector function, whereas pathway-directed or stromal-targeted strategies may be more appropriate when adaptive signaling or spatial exclusion predominates. Ultimately, effective treatment of resistant HER2-positive breast cancer will require rational selection and sequencing of therapies that simultaneously address tumor-cell dependence and the dominant microenvironmental mechanisms sustaining resistance. These hypotheses require prospective validation but provide a framework for the next generation of resistance-focused trials.

## Data Availability

Not applicable.

## Abbreviation

**Abbreviation**	**Full term**
HER2	Human epidermal growth factor receptor 2
TME	Tumor microenvironment
CAFs	Cancer-associated fibroblasts
TAMs	Tumor-associated macrophages
Tregs	Regulatory T cells
MDSCs	Myeloid-derived suppressor cells
ECM	Extracellular matrix
DTP	Drug-tolerant persister
NK	Natural killer
ADCC	Antibody-dependent cellular cytotoxicity
TILs	Tumor-infiltrating lymphocytes
HR	Hormone receptor
IL	Interleukin
TNFα	Tumor necrosis factor alpha
sTNFα	Soluble tumor necrosis factor alpha
ROS	Reactive oxygen species
VEGF	Vascular endothelial growth factor
MUC4	Mucin 4
EZH2	Enhancer of zeste homolog 2
MHC	Major histocompatibility complex
EMT	Epithelial–mesenchymal transition
CSCs	Cancer stem cells
PI3K	Phosphoinositide 3-kinase
AKT	Protein kinase B
mTOR	Mechanistic target of rapamycin
mTORC1	Mechanistic target of rapamycin complex 1
MAPK	Mitogen-activated protein kinase
ERK	Extracellular signal-regulated kinase
JAK	Janus kinase
STAT3	Signal transducer and activator of transcription 3
NF-κB	Nuclear factor kappa B
FGFR	Fibroblast growth factor receptor
HER3	Human epidermal growth factor receptor 3
IGF-1	Insulin-like growth factor 1
IGF-1R	Insulin-like growth factor 1 receptor
NRG1	Neuregulin 1
ADC	Antibody–drug conjugate
T-DM1	Trastuzumab emtansine
T-DXd	Trastuzumab deruxtecan
TKI	Tyrosine kinase inhibitor
PFS	Progression-free survival
ORR	Objective response rate
pCR	Pathological complete response
PD-L1	Programmed death-ligand 1
PD-1	Programmed cell death protein 1
CTLA-4	Cytotoxic T-lymphocyte-associated protein 4
TIGIT	T-cell immunoreceptor with immunoglobulin and ITIM domains
SIRPα	Signal regulatory protein alpha
CCR4	C-C chemokine receptor type 4
CXCL	C-X-C motif chemokine ligand
CCL	C-C motif chemokine ligand
FOXP3	Forkhead box P3
PDPN	Podoplanin
scRNA-seq	Single-cell RNA sequencing
LC–MS/MS	Liquid chromatography–tandem mass spectrometry
